# Auditory conflict and congruence in frontotemporal dementia

**DOI:** 10.1016/j.neuropsychologia.2017.08.009

**Published:** 2017-09

**Authors:** Camilla N. Clark, Jennifer M. Nicholas, Jennifer L. Agustus, Christopher J.D. Hardy, Lucy L. Russell, Emilie V. Brotherhood, Katrina M. Dick, Charles R. Marshall, Catherine J. Mummery, Jonathan D. Rohrer, Jason D. Warren

**Affiliations:** a Dementia Research Centre, UCL Institute of Neurology, University College London, London, United Kingdom; b London School of Hygiene and Tropical Medicine, University of London, London, United Kingdomt

**Keywords:** Auditory, Conflict, Congruence, Emotion, Frontotemporal dementia, Semantic dementia

## Abstract

Impaired analysis of signal conflict and congruence may contribute to diverse socio-emotional symptoms in frontotemporal dementias, however the underlying mechanisms have not been defined. Here we addressed this issue in patients with behavioural variant frontotemporal dementia (bvFTD; n = 19) and semantic dementia (SD; n = 10) relative to healthy older individuals (n = 20). We created auditory scenes in which semantic and emotional congruity of constituent sounds were independently probed; associated tasks controlled for auditory perceptual similarity, scene parsing and semantic competence. Neuroanatomical correlates of auditory congruity processing were assessed using voxel-based morphometry. Relative to healthy controls, both the bvFTD and SD groups had impaired semantic and emotional congruity processing (after taking auditory control task performance into account) and reduced affective integration of sounds into scenes. Grey matter correlates of auditory semantic congruity processing were identified in distributed regions encompassing prefrontal, parieto-temporal and insular areas and correlates of auditory emotional congruity in partly overlapping temporal, insular and striatal regions. Our findings suggest that decoding of auditory signal relatedness may probe a generic cognitive mechanism and neural architecture underpinning frontotemporal dementia syndromes.

## Highlights

• Socioemotional changes in frontotemporal dementia may reflect altered signal decoding.
• To address this, we varied semantic and emotional signal relatedness in sound scenes.
• Frontotemporal dementia patients had impaired semantic and emotional scene decoding.
• These deficits correlated with atrophy of distributed cortico-subcortical regions.
• Signal processing approaches may help deconstruct complex dementia phenotypes.

## 1 Introduction

Natural sensory environments or scenes often convey a cacophonous mixture of signals. Successful decoding of such scenes depends on resolution of the sensory mixture to enable a coherent behavioural and emotional response. Competing or conflicting signals present an important challenge to this enterprise. Signal conflict (simultaneous activation of incompatible or divergent representations or associations, Botvinick et al., 2001) often requires modification of behavioural goals; an appropriate behavioural response depends on detecting the salient signal mismatch and decoding its semantic and emotional significance. Equally, accurate determination of signal similarities and congruence is essential to establish regularities in the environment that can guide future adaptive behaviours. Analysis of signal ‘relatedness’ (conflict versus congruence) and conflict resolution are integral to complex decision making and emotional responses, particularly in social contexts (Chan et al., 2012, Clark et al., 2015b, Moran et al., 2004).

In neurobiological terms, behavioural responses to sensory signal relatedness reflect the operation of hierarchically organised generative models (Cohen, 2014, Nazimek et al., 2013, Silvetti et al., 2014). These models form predictions about the environment based on current and previous sensory experience, detect unexpected or ‘surprising’ events as prediction errors and adjust behavioural output to minimise those errors (Friston, 2009, Moran et al., 2004). The underlying neural computations engage large-scale brain networks: these networks encompass posterior cortical areas that parse sensory traffic into component objects; medial fronto-parietal cortices that direct and control attention and the detection of salient sensory events according to behavioural context; antero-medial temporal areas that store previously learned knowledge and schemas about sensory objects and regularities; insular and prefrontal cortices that implement and assess violations in rule-based algorithms; and striatal and other subcortical structures that code emotional and physiological value (Christensen et al., 2011, Cohen, 2014, Dieguez-Risco et al., 2015, Dzafic et al., 2016, Gauvin et al., 2016, Groussard et al., 2010, Henderson et al., 2016, Jakuszeit et al., 2013, Klasen et al., 2011, Merkel et al., 2015, Michelon et al., 2003, Nazimek et al., 2013, Remy et al., 2014, Ridderinkhof et al., 2004, Rosenbloom et al., 2012, Silvetti et al., 2014, Watanabe et al., 2014), Within this distributed circuitry, separable mechanisms have been identified for the processing of semantic and affective congruence (Dieguez-Risco et al., 2015) and for elementary versus more abstract levels of incongruity decoding (Paavilainen, 2013).

On clinical as well as neuroanatomical grounds, abnormal processing of conflict and congruence is a candidate generic mechanism of disease phenotypes in the frontotemporal dementias (Warren et al., 2013). These diseases collectively constitute an important cause of young onset dementia and manifest clinically with diverse deficits of semantic, emotional and social signal decoding, particularly in the syndromes of behavioural variant frontotemporal dementia (bvFTD) and semantic dementia (SD) (Downey et al., 2015, Fumagalli and Priori, 2012, Irish et al., 2014, Kipps et al., 2009, Piwnica-Worms et al., 2010, Snowden et al., 2003, St Jacques et al., 2015, Warren et al., 2013). Although bvFTD is defined by early, prominent behavioural and emotional impairments while SD is defined by progressive, pan-modal impairment of semantic memory, these two syndromes substantially overlap, both clinically and neuroanatomically (Gorno-Tempini et al., 2011, Hodges and Patterson, 2007, Rascovsky et al., 2011, Warren et al., 2013). Key deficits in both syndromes may reflect impaired integration of context and perspective taking (Ibanez and Manes, 2012). Inability to reconcile different perspectives may contribute more specifically to loss of empathy and theory of mind (Baez et al., 2014, Irish et al., 2014, Kipps et al., 2009), reduced self-awareness (Sturm et al., 2013), aberrant resolution of moral and social dilemmas (Carr et al., 2015, Eslinger et al., 2007) and abnormally polarised behaviours (Clark and Warren, 2016). Defective recruitment of stored social and semantic schemas may reduce adherence to social regularities (Zahn et al., 2007) while impaired ability to modify behaviour in response to ‘surprising’ events may contribute to dysfunctional reward seeking and valuation (Dalton et al., 2012, Perry et al., 2014). Abnormal conflict monitoring has been documented early in bvFTD (Krueger et al., 2009) and it remains uncertain as to what extent this reflects more general executive dysfunction (Seer et al., 2015). Neuroanatomically, the candidate network substrates for processing signal relatedness overlap key areas of disease involvement in bvFTD and SD (Fletcher and Warren, 2011, Hodges and Patterson, 2007, Perry et al., 2014, Warren et al., 2013). Despite much clinical and neurobiological interest, fundamental or generic models and mechanisms that can capture the clinical and neuroanatomical heterogeneity of frontotemporal dementia are largely lacking. There would be considerable interest in identifying a model system that reflects important clinical deficits in these diseases, while at the same time allowing those deficits to be more easily understood, measured and tracked, with a view to the development and evaluation of therapies.

Nonverbal sound is one such attractive model sensory system, with particular resonance for frontotemporal dementia and the potentially unifying theme of abnormal conflict and congruence signalling. Signal prediction and detection of violated predictions are likely to be intrinsic to the analysis of auditory scenes, in line with the commonplace observation that sound events (such as ‘things that go bump in the night’) are often ambiguous and require active contextual decoding to prepare an appropriate behavioural response (Fletcher et al., 2016). The requirements for disambiguating competing sound sources, tracking of sound sources dynamically over time and linking sound percepts to stored semantic and emotional associations all impose heavy computational demands on neural processing mechanisms. Moreover, the fronto-temporo-parietal and subcortical brain networks that instantiate these mechanisms are selectively targeted by the disease process in frontotemporal dementias (Hardy et al., 2017, Warren et al., 2013). One might therefore predict abnormalities of sound signal decoding in these diseases and indeed, a range of a auditory deficits have been described, ranging from impaired electrophysiological responses to acoustic oddballs (Hughes et al., 2013) to complex cognitive and behavioural phenotypes (Downey et al., 2015, Fletcher et al., 2015a, Fletcher et al., 2016, Fletcher et al., 2015b, Hardy et al., 2017). Many of these phenotypic features might arise from impaired integration of auditory signals and impaired processing of signal mismatch. However, the relevant cognitive and neuroanatomical mechanisms have not been defined.

Here we addressed the processing of signal conflict and congruence in auditory environments in two canonical syndromes of frontotemporal dementia, bvFTD and SD relative to healthy older individuals. We designed a novel behavioural paradigm requiring decisions about auditory ‘scenes’, each comprising two competing sound sources in which the congruity or incongruity of the sources was varied along semantic (identity relatedness) and affective (emotional relatedness) dimensions independently. We constructed ‘model’ scenes that would simulate naturalistic processing of the kind entailed by real world listening while still allowing explicit manipulation of the stimulus parameters of interest. The stimulus dimensions of semantic and emotional congruity were anticipated to be particularly vulnerable to the target syndromes, based on an extensive clinical and neuropsychological literature in auditory and other cognitive domains (Hardy et al., 2017, Hodges and Patterson, 2007, Warren et al., 2013). Structural neuroanatomical associations of experimental task performance were assessed using voxel-based morphometry in the patient cohort.

We hypothesised firstly that both bvFTD and SD (relative to healthy older individuals) would be associated with impaired detection and affective valuation of auditory signal relatedness, given that these syndromes show qualitatively similar semantic and affective deficits when required to integrate information from social and other complex auditory signals (Downey et al., 2015, Fletcher et al., 2015a, Fletcher et al., 2016, Fletcher et al., 2015b, Hodges and Patterson, 2007, Rascovsky et al., 2007, Warren et al., 2013). We further hypothesised that these deficits would be evident after taking into account background auditory perceptual and general cognitive competence. We anticipated that the decoding of both semantic and affective auditory relatedness would have a neuroanatomical correlate in anterior temporal and insula cortical ‘hubs’ for processing signal salience based on prior expectations (Christensen et al., 2011, Groussard et al., 2010, Merkel et al., 2015, Nazimek et al., 2013, Remy et al., 2014, Watanabe et al., 2014). Finally, we hypothesised that the analysis of auditory semantic congruence would have an additional correlate in fronto-parietal cortices previously linked to processing of rule violations and conflict resolution (Chan et al., 2012, Groussard et al., 2010, Henderson et al., 2016, Jakuszeit et al., 2013, Paavilainen, 2013, Remy et al., 2014, Ridderinkhof et al., 2004, Rosenbloom et al., 2012, Strelnikov et al., 2006); while the analysis of auditory emotional congruence would have an additional subcortical correlate in striatal and mesial temporal structures previously linked to the processing of emotional congruence and associated reward value (Dzafic et al., 2016, Klasen et al., 2011, Schultz, 2013).

## 2 Methods

### 2.1 Participant groups

Twenty-nine consecutive patients fulfilling current consensus criteria for bvFTD ((Rascovsky et al., 2011); n = 19, mean age 64 years (standard deviation 7.2 years), three female) or SD ((Gorno-Tempini et al., 2011); n = 10, mean age 66.2 (6.3) years, four female) were recruited via a tertiary specialist cognitive clinic; 20 healthy older individuals (mean age 68.8 (5.3) years, 11 female) with no history of neurological or psychiatric illness also participated. None of the participants had a history of clinically relevant hearing loss. Demographic and general neuropsychological characteristics of the study cohort are summarised in Table 1. Syndromic diagnoses in the patient groups were corroborated with a comprehensive general neuropsychological assessment (Table 1). Genetic screening of the whole patient cohort revealed pathogenic mutations in eight patients in the bvFTD group (five *MAPT,* three *C9orf72*); no other pathogenic mutations were identified. CSF examination was performed in six patients with sporadic bvFTD and in five patients with SD: profiles of CSF neurodegeneration markers in these cases provided no evidence for underlying AD pathology based on local laboratory reference ranges (i.e., no patient had total CSF tau: beta-amyloid_1–42_ ratio > 1). In total 14 patients in the bvFTD group had either a pathogenic mutation, consistent CSF neurodegenerative markers or both. Clinical brain imaging (MRI or CT) revealed variably asymmetric but compatible profiles of atrophy across the patient cohort (Table 1). No brain images showed a significant cerebrovascular burden.

**Table 1:** General characteristics of participant groups.

**Characteristic**	**Healthy controls**	**bvFTD**	**SD**
**General**			
No. (m:f)	9:11	**16:3**	6:4
Handedness (R:L)	17:3	17:2	9:1
Age (yrs)	69 (5.3)	64 (7.2)	66 (6.3)
Education (yrs)	16.4 (2.0)	15.1 (2.8)	15.6 (2.6)
MMSE (/30)	29 (1.4)	24.3 (4.5)	21.3 (6.3)
Symptom duration (yrs)	N/A	8.1 (6.3)	5.3 (2.9)
**Neuroanatomical**			
Brain MRI atrophy:			
Temporal predom L: symm: predom R	N/A	0: 4: 7	9:0: 1
Frontotemporal symmetric	N/A	8	0
**Neuropsychological**			
***General intellect: IQ***			
WASI verbal IQ	126 (7.2)	**84 (22.2)**	**75 (17.0)**
WASI performance IQ	124 (9.6)	**102 (20.7)**	**106 (21.9)**
***Executive skills***			
WASI Block Design (/71)	45.4( 12.1)	32.5 (18.1)	36.8 (20.7)
WASI Matrices (/32)	26.5 (2.9)	**18.4 (9.0)**	**19.8 (9.8)**
WMS-R digit span forward (/12)	9.2 (2.2)	8.6 (2.8)	8.2 (2.6)
WMS-R digit span reverse (/12)	7.8 (2.2)	**5.8 (2.5)**	**6.0 (3.0)**
D-KEFS Stroop colour (s)*	32.0 (6.3)	**46.9 (15.8)**	**60.7 (31.9)**
D-KEFS Stroop word (s)*	23.7 (5.9)	32.2 (12.3)	36.2 (22.1)
D-KEFS Stroop interference (s)*	58.1 (17.0)	**88.4 (31.3)**	88.3 (48.8)
Letter fluency (F: total)	17.4 (4.4)	**7.7 (5.4)**	**10.0 (4.8)**
Category fluency (animals: total)	25.3 (5.0)	**10.5 (6.8)**	**6.2 (5.1)**
Trails A (s)	32.5 (7.4)	**59.8 (34.4)**	**52.2 (17.8)**
Trails B (s)	67.1 (18.0)	**158 (81)**	**154 (112)**
WAIS-R Digit Symbol (total)	54.9 (11.1)	**35.6 (13.4)**	**39.7 (13.9)**
***Semantic memory***			
BPVS (/150)	149 (1.1)	**123 (33.6)**	**95 (47.4)**
Synonyms concrete(/25)	24.1 (0.76)	N/A	**16.3 (3.5)**
Synonyms abstract(/25)	24.3 (0.91)	N/A	**18.8 (3.1)**
***Language skills***			
WASI Vocabulary (/80)	72.7 (3.27)	**39.7 (21.2)**	**31.8 (19.9)**
WASI Similarities (/48)	41.5 (2.9)	**23 (12.0)**	**17.2 (11.0)**
GNT (/30)	26.6 (2.3)	**12.3 (9.6)**	**3.4 (6.1)**†
NART (total correct/50)	43.2 (4.9)	**30.4 (10.0)**	**19.2 (14.2)**†
***Episodic memory***			
RMT words (/50)	49.4 (0.9)	**37.1 (8.9)**	**37 (6.7)**
RMT faces (/50)	44.7 (3.6)	**34.5 (7.8)**	**32.3 (7.0)**
Camden PAL (/24)	20.5 (3.2)	**10.7 (7.5)**	**3.8 (3.9)**†
***Posterior cortical skills***			
GDA (/24)	14.8 (5.6)	**8.6 (6.8)**	11.1 (9.0)
VOSP Object Decision (/20)	18.9 (1.6)	**16.3 (2.6)**	**16.3 (4.3)**

Mean (standard deviation) scores are shown unless otherwise indicated; maximum scores are shown after tests (in parentheses). Bold denotes significantly different (p < 0.05) to the healthy control group; † significant difference between disease groups. *Delis-Kaplan Executive Function System versions of the traditional Stroop tests were used. Each condition comprises a 10 (column) × 5 (row) grid of targets and the participant is required to name all the targets from left to right in each row. In the ‘colour’ condition, the participant must correctly name each patch of colour in the grid (“red/ blue/ green”). In the ‘word’ condition, they must correctly read each word in the grid (“red/ blue/ green”). In the ‘interference’ condition, they must correctly identify the colour of the ink that each word is written in; this will be incongruous with the written word (e.g. the correct response to the word “red” printed in green ink is “green”). Scores here denote time taken to complete each grid in seconds. BPVS, British Picture Vocabulary Scale (Dunn et al., 1982); bvFTD, behavioural variant frontotemporal dementia; Category fluency for animal category and letter fluency for the letter F in one minute (Gladsjo et al., 1999); GDA, Graded Difficulty Arithmetic (Jackson and Warrington, 1986); GNT, Graded Naming Test (McKenna and Warrington, 1983); MMSE, Mini-Mental State Examination score (Folstein et al., 1975); N/A, not assessed; NART, National Adult Reading Test (Nelson, 1982); PAL, Paired Associate Learning test (Warrington, 1996); predom L/R, predominantly left / right temporal lobe atrophy; RMT, Recognition Memory Test (Warrington, 1984); symm, symmetric (temporal lobe) atrophy; Synonyms, Single Word Comprehension: A Concrete and Abstract Word Synonyms Test (E.K. Warrington et al., 1998); SD, semantic dementia; Stroop D-KEFS, Delis Kaplan Executive Function System (Delis et al., 2001); Trails-making task based on maximum time achievable 2.5 min on task A, 5 min on task B (Lezak et al., 2004); VOSP, Visual Object and Spatial Perception Battery (E.K. Warrington and James, 1991); WAIS-R, Wechsler Adult Intelligence Scale‐-Revised (D Wechsler, 1981); WASI, Wechsler Abbreviated Scale of Intelligence (D. Wechsler, 1997); WMS digit span (Wechsler, 1987).

The study was approved by the local institutional ethics committee and all participants gave informed consent in accordance with the guidelines of the Declaration of Helsinki.

### 2.2 Experimental design

#### 2.2.1 Auditory scene tests

**Fig. 1:**
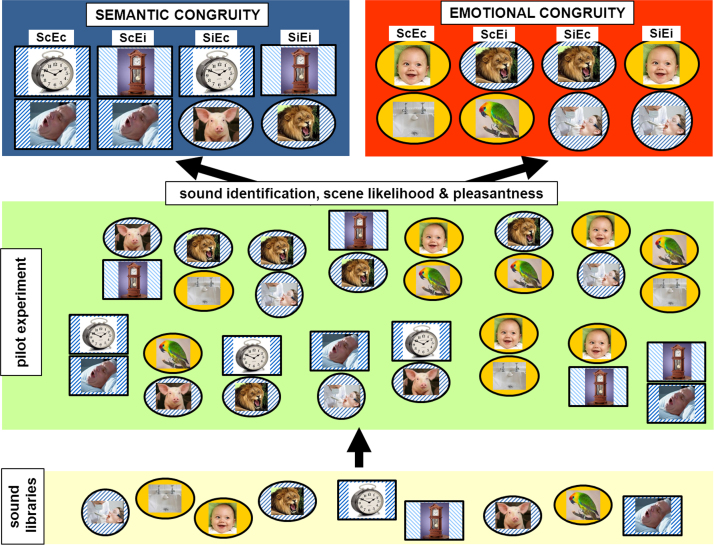
Procedure for creating auditory scene congruity tests. The diagram summarises the key steps we followed in preparing the auditory semantic and emotional congruity tests in the main experiment. An initial search of sound libraries (bottom panel; listed in Supplementary Material on-line) identified 62 sounds drawn from the broad categories of human and animal vocalisations, natural environmental noises and artificial noises (machinery and tools), of which a subset of nine sounds are represented pictorially here (from left to right, dentist's drill, splashing water, baby laughing, lion, alarm clock, grandfather clock, pig, bird chirping, snoring). These sounds were superimposed digitally as pairs into scenes (see Supplementary Material on-line) with fixed duration and average loudness. In the pilot experiment (middle panel; details in Supplementary Material on-line), the 62 constituent sounds individually were assessed for identifiability and pleasantness; and 193 sound scenes (composed from paired sounds) were assessed for likelihood and pleasantness of the combination. Auditory scene stimuli in the final semantic and emotional congruity tests (top panels; 30 trials in semantic congruity test, 40 trials in emotional congruity test) comprised the following conditions: **ScEc**, semantically congruous, emotionally congruous; **ScEi**, semantically congruous, emotionally incongruous; **SiEc**, semantically incongruous, emotionally congruous; **SiEi**, semantically incongruous, emotionally incongruous (here, semantic relatedness is coded using sound icon shape and emotional relatedness using sound icon shading). These final scene stimuli met inclusion criteria established from the pilot data (details in Supplementary Material on-line): all individual constituent sounds met a consensus identifiability criterion and in addition, scenes in the final semantic congruity test met condition-specific likelihood criteria while scenes in the final emotional congruity test met condition-specific pleasantness criteria. For each test, the ‘nuisance’ congruity parameter (emotional congruity in the semantic congruity test; semantic congruity in the emotional congruity test) was also controlled within a narrow range across conditions.

We created auditory scene stimuli based on overlaid pairs of sounds (examples in Supplementary Material on-line) in which the congruity of the two sounds was varied independently along two dimensions; semantic (whether the sounds would be likely or unlikely to occur together) and emotional (whether the sounds had similar or contrasting affective valence). The procedure we followed in preparing the auditory scene congruity tests is diagramed in Fig. 1.

Individual sounds were obtained from on-line digital databases to sample semantic categories of human nonverbal sounds, animal sounds, natural environmental noises and artificial noises (machinery and tools).

Pairs of sounds were superimposed using Goldwave® software, further details of stimulus synthesis are in Supplementary Material on-line. The resulting auditory ‘scenes’ comprised four conditions (balanced for their constituent sounds) in a factorial matrix; semantically congruous – emotionally congruous, ScEc (e.g., alarm clock- snoring); semantically incongruous – emotionally congruous, SiEc (e.g., alarm clock – pig grunting); semantically congruous – emotionally incongruous (e.g., chiming clock – snoring); semantically incongruous – emotionally incongruous, SiEi (e.g., chiming clock – roaring lion). Auditory scene stimuli were edited to fixed duration (8 s) and mean intensity level. Based on an initial pilot experiment in healthy older individuals (details in Supplementary Material on-line), a final set of 60 auditory scene stimuli (comprising combinations of 43 individual sounds) was selected from a larger set of 193 candidate auditory scenes, using criteria of > 80% correct identification of both constituent sounds in each scene and rated likelihood and pleasantness of the scene (the sound combination) by the healthy pilot group.

The final auditory scene stimuli were arranged to create two tests, each incorporating the four sound conditions (ScEc, SiEc, ScEi, SiEi), but requiring a decision on either the semantic congruity or the emotional congruity of the sound scenes. A forced-choice response procedure was used in both tests. Stimuli for each test are listed in Tables S1 and S2 in Supplementary Material on-line. In constructing each test, pilot control ratings were used to classify sound pairs for the parameter of interest while balancing across conditions for the other, nuisance parameter. For the semantic congruity test, likelihood of co-occurrence was the relevant parameter and pleasantness discrepancy was the nuisance parameter; for the emotional congruity test, these roles were reversed. An auditory scene was included in the final stimulus set if i) both constituent sounds were identified correctly by > 80% of the pilot healthy control group and ii) the scene overall met an additional congruity criterion, based on pilot group ratings (for the semantic congruity test, rated likelihood of co-occurrence of the two sounds and for the emotional congruity test, rated pleasantness discrepancy of the two sounds). In addition, scenes were selected such that each test was balanced wherever feasible for the ‘nuisance’ congruity parameter (for the semantic congruity test, the pleasantness discrepancy rating; for the emotional congruity test, the likelihood rating) and the individual sounds represented across conditions; and for the relative proportions of pleasant and unpleasant sound pairs comprising the congruous conditions. The semantic congruity test comprised 30 trials (15 congruous, 15 incongruous); the participant's task on each trial was to decide whether or not the sounds in the scene would usually be heard together. The emotional congruity test comprised 40 trials (20 congruous, 20 incongruous); the participant's task on each trial was to decide whether the sounds in the scene were both pleasant, both unpleasant or a mixture of pleasant and unpleasant. In addition, on each trial in the emotional congruity test the participant rated the overall pleasantness of the auditory scene (the sound combination) on a Likert scale (1 = very unpleasant, to 5 = very pleasant).

#### 2.2.2 Control tests

In order to interpret participants’ performance on the auditory scene tests, we created control tests to probe auditory perceptual similarity processing, auditory scene analysis and semantic knowledge of individual sounds.

In the perceptual similarity control test, we assessed each participant's ability to perceive acoustic similarity and variation between two sounds. Concatenated sounds were presented such that the sequence of sounds either comprised a single sound source or two sound sources of a single kind (for example, a small dog and a large dog). The individual acoustic tokens comprising the sequence were always varied (for example, different barks from the same small or large dog). Thirty trials (15 containing a change in source, 15 with no change in source) sampling different semantic categories were presented; the task on each trial was to decide if the thing making the sound changed or remained the same. This task served as a control both for the perceptual analysis of constituent sounds and the decision-making procedure used in the tests of semantic and emotional congruity judgment.

In the auditory scene control test, we assessed each participant's ability to parse superimposed sounds. We adapted an existing test (Golden et al., 2015) requiring identification of a personal name (e.g. ‘Robert’) spoken over multi-talker babble. Twenty trials were presented; the task on each trial was to identify the spoken name.

In the auditory semantic (sound identification) control test, we assessed each participant's ability to identify and affectively evaluate individual sounds. All 43 constituent sounds composing the auditory scene stimulus set were presented individually; the task on each trial was to match the sound to one of three pictures representing the sound source (e.g., duck), a closely semantically related foil (e.g., gull) and a distantly semantically related foil (e.g., train). In addition, the participant was asked to rate the pleasantness of each sound on a Likert scale (1 = very unpleasant, to 5 = very pleasant).

### 2.3 General experimental procedure

All stimuli were delivered from a notebook computer running MATLAB® via headphones (Audio-Technica®) at a comfortable listening level for each participant in a quiet room. Within each test, trials representing each condition were presented in randomised order. Participants were first familiarised with each test using practice examples (not administered in the subsequent test) to ensure they understood the task instructions and were able to comply reliably. Participant responses were recorded for offline analysis. During the tests no feedback was given about performance and no time limits were imposed.

### 2.4 Analysis of behavioural data

All behavioural data were analysed using Stata12®. Demographic characteristics and general neuropsychological data were compared between participant groups using (for categorical variables) Fisher's exact test or (for continuous variables) either two sample *t*-tests or Wilcoxon rank sum tests, where assumptions for the *t*-test were materially violated (for example, due to skewed data distribution).

On the perceptual similarity, auditory scene control and auditory semantic control tests, the proportion of correct responses was analysed using a logistic regression model owing to a binary outcome (correct / incorrect), with robust standard errors to account for clustering by participant. Mean overall pleasantness ratings of individual sounds on the auditory semantic control test were compared between participant groups using linear regression with bias corrected, accelerated confidence intervals from 2000 bootstrap replications due to the skewed (non-normal) distribution of the data. In each model, participant group was included as a categorical predictor and age, gender and reverse digit span (an index of executive and auditory working memory function) were included as (where appropriate, mean-centred) nuisance covariates.

In order to interpret the processing of auditory scene congruity in the main experiment, we wished to take into account whether the constituent sounds in a scene were identified correctly. Data for the semantic and emotional congruity decision tasks on auditory scene stimuli were pre-processed using data from the auditory semantic (sound identification) control test. For each participant, congruity decisions were scored only for those scene stimuli containing sounds that were both identified correctly when presented in isolation in the auditory semantic control test. Analyses were therefore based on different subsets of the scene stimuli in each participant group (numbers of stimuli included in these subanalyses are indicated in Table S3 in Supplementary Material on-line; note that all participants heard the same full set of stimuli). This analysis strategy allowed us to assess auditory scene semantic and affective processing independently of more elementary auditory semantic knowledge about particular sounds. As the subset of scene stimuli included in the final analysis could therefore potentially vary between individual participants and groups, scene parameters of likelihood and pleasantness (based on pilot data) were assessed to ensure there was no systematic bias that might have altered the effective difficulty of the stimulus subset for a particular participant group; this post hoc analysis revealed that the likelihood and pleasantness of the scene stimuli included in the final analysis were similar across participant groups (details in Supplementary Material on-line). For the auditory scene congruity tests, the proportion of correct responses for each test was compared between participant groups using logistic regression on the binary outcome variable (correct / incorrect) and allowing for a clustering of responses by individual. Participant group was included as a categorical predictor in the model and (where appropriate, mean-centred) nuisance covariates of age, gender, reverse digit span and scores on the perceptual similarity and auditory scene control tasks were also included. Although we did not anticipate differential impairment according to the congruity of the stimuli, this was formally tested by fitting a second logistic model with two-way interaction between participant and congruity condition, including the same nuisance covariates.

Auditory scene pleasantness rating data in the emotional congruity test were compared between participant groups using a multiple linear regression model that allowed us to distinguish the effect of combining sounds into scenes from individual sound pleasantness. Overall auditory scene pleasantness might be biased by particular, strongly emotional constituent sounds and the extent of any such bias might itself be susceptible to disease. The model therefore incorporated separate terms for participant group, each participant's own (potentially idiosyncratic) pleasantness ratings of both sounds individually and the interaction of the sounds in an auditory scene. This model allowed us to go beyond any abnormal rating of individual sound pleasantness in the disease groups, to assess group differences in the rating of sound combinations. To account for violated normality assumptions, the analysis used bias corrected, accelerated confidence intervals based on 2000 bootstrap replications.

In separate post hoc analyses, for each patient group separately we assessed for correlations between key cognitive measures of interest using Spearman's correlation coefficient. Specifically, we assessed the extent of any correlation between semantic and emotional scene congruity performance; between semantic scene congruity and individual sound recognition performance; and between congruity decisions and performance on a standard test of nonverbal executive function (WASI Matrices), a standard index of semantic competence (British Picture Vocabulary Scale (BPVS) score) and a surrogate measure of disease severity (Mini-Mental State Examination (MMSE) score)

A threshold p < 0.05 was accepted as the criterion for statistical significance in all analyses.

### 2.5 Brain image acquisition and pre-processing

Volumetric brain MRI data were acquired for 27 patients (18 bvFTD, nine SD) on a Siemens Trio 3Tesla MRI scanner using a 32-channel phased array head-coil and a sagittal 3-D magnetization prepared rapid gradient echo T1-weighted volumetric sequence (echo time/repetition time/inversion time = 2.9/2200/900 ms, dimensions 256 × 256 × 208, voxel size 1.1 × 1.1 × 1.1 mm). Volumetric brain images were assessed visually in all planes to ensure adequate coverage and to exclude artefacts or significant motion. Pre-processing of patient brain MR images was performed using the Segment routine and the DARTEL toolbox of SPM12 (Ashburner, 2007, fil.ion.ucl.ac.uk/spm/, 1994–2013). Normalisation, segmentation and modulation of grey and white matter images used default parameter settings, with a smoothing Gaussian kernel of full-width-at-half-maximum 6 mm. Smoothed segments were warped into MNI space using the “Normalise to MNI” routine. In order to adjust for individual differences in global grey matter volume during subsequent analysis, total intracranial volume (TIV) was calculated for each participant by summing grey matter, white matter and cerebrospinal fluid volumes following segmentation of all three tissue classes. A study-specific mean brain image template, for displaying results, was created by warping all bias-corrected native space whole-brain images to the final DARTEL template in MNI space and calculating the average of the warped brains. To help protect against voxel drop-out due to marked local regional atrophy, a customised explicit brain mask was made based on a specified ‘consensus’ voxel threshold intensity criterion (Ridgway et al., 2009), whereby a particular voxel was included in the analysis if grey matter intensity at that voxel was > 0.1 in > 70% of participants (rather than in all participants, as with the default SPM mask). The mask was applied to the smoothed grey matter segments prior to statistical analysis.

### 2.6 Voxel-based morphometry analysis

Using the framework of the general linear model, multiple regression was used to examine associations between voxel intensity (grey matter volume) and behavioural variables of interest over the combined patient cohort. In separate design matrices, voxel intensity was modelled as a function of participant scores on the semantic and emotional congruity tasks and the perceptual similarity, auditory scene and auditory semantic control tasks. In all models, age, gender, TIV, syndromic group and reverse digit span were included as nuisance covariates. For each model, we assessed both positive and negative (inverse) grey matter associations of the behavioural variable of interest. Statistical parametric maps were thresholded at two levels of significance: p < 0.05 after family-wise error (FWE) correction for multiple voxel-wise comparisons over the whole brain; and p < 0.05 after FWE correction for multiple voxel-wise comparisons within defined regions of interest based on our prior anatomical hypotheses.

The anatomical regions used for small volume correction (displayed in Fig. S1 in Supplementary Material on-line) covered key areas in both hemispheres that have been implicated in nonverbal sound and incongruity processing in the healthy brain, stratified for the contrasts of interest. These regions of interest comprised: for all contrasts, a posterior temporo-parietal region combining posterior superior temporal gyrus, lateral inferior parietal cortex and posterior medial cortex (previously implicated in auditory scene parsing and incongruity processing: Chan et al., 2012, Groussard et al., 2010, Gutschalk and Dykstra, 2013, Pinhas et al., 2015, Zundorf et al., 2013); and for the contrasts based on semantic and/or congruity processing, additional regions combining anterior and medial temporal lobe anterior to Heschl's gyrus, combining insula and inferior frontal gyrus (previously implicated in auditory semantic and rule decoding: Christensen et al., 2011, Groussard et al., 2010, Henderson et al., 2016, Jakuszeit et al., 2013, Merkel et al., 2015, Nazimek et al., 2013, Remy et al., 2014, Watanabe et al., 2014, Zahn et al., 2007), and anterior cingulate cortex and striatum (previously implicated in salience, emotion and reward evaluation: Ridderinkhof et al., 2004, Rosenbloom et al., 2012, Schultz, 2013, Watanabe et al., 2014). Regions were derived from the Oxford-Harvard brain maps (Desikan et al., 2006) in FSLview (Jenkinson et al., 2012) and edited using MRIcron (mccauslandcenter.sc.edu/mricro/mricron/) to conform to the study template (participant mean) brain image.

As a reference for interpreting the correlative analysis, we conducted an additional, separate analysis to assess disease-related grey matter atrophy profiles in each of the patient groups, comparing patients’ brain MR images with brain images acquired in the healthy control group using the same scanning protocol. Groups were compared using voxel-wise two-sample *t*-tests, including covariates of age, gender, and TIV. Statistical parametric maps of brain atrophy were thresholded leniently (p < 0.01 uncorrected over the whole brain volume) in order to more fully delineate the profile of atrophy in each patient group.

## 3 Results

### 3.1 General characteristics of participant groups

The participant groups did not differ for age (p = 0.07) or educational background (p = 0.25) and the patient groups did not differ in mean symptom duration (p = 0.32). Gender distribution differed significantly between groups, males being significantly over-represented in the bvFTD group relative to the healthy control group (p = 0.019); gender was incorporated as a nuisance covariate in all subsequent analyses. The patient groups showed the anticipated profiles of general neuropsychological impairment (Table 1).

### 3.2 Experimental behavioural data

#### 3.2.1 Auditory control task performance

Performance profiles of participant groups on the perceptual similarity, auditory scene and auditory semantic control tests are summarised in Table 2. On the perceptual similarity control task, the bvFTD group performed significantly worse than both the healthy control group (p < 0.0001]) and the SD group (p = 0.027]), whereas the SD group performed similarly to healthy controls (p = 0.153]). On the auditory scene control task, both patient groups performed significantly worse than the healthy control group (both p < 0.001]). There was no significant performance difference between patient groups (p = 0.96]). On the auditory semantic control (sound identification) task, both patient groups performed significantly worse than the healthy control group (both p < 0.001). There was no significant performance difference between patient groups (p = 0.92). Overall pleasantness ratings of individual sounds did not differ significantly for either patient group versus healthy controls (bvFTD, β = 0.08 [95% confidence interval (CI) −0.33 to 0.45, p > 0.05]; SD, β = 0.44 [95% CI −0.10 to 1.00, p > 0.05]) nor between patient groups (β = 0.36 [95% CI −0.22 to 0.88, p > 0.05]). Inspection of individual sound pleasantness ratings suggests that affective valuation of particular constituent sounds was similar between participant groups (see Table S4 in Supplementary Material on-line); this factor is therefore unlikely to have driven any group differences in the affective processing of sounds combined as scenes.

**Table 2:** Performance of patient groups on auditory tasks versus healthy controls.

**Test**	**bvFTD**	**SD**
**CONTROL TASKS**		
**Perceptual similarity**	**0.32 (0.19–0.54)**	0.65 (0.36–1.17)
**Auditory scene analysis**	**0.11 (0.05–0.29)**	**0.10 (0.04–0.26)**
**Sound identification**	**0.03 (0.008–0.12)**	**0.04 (0.01–0.19)**
**AUDITORY SCENE CONGRUITY**		
**Semantic**		
***ScEc***	**0.35 (0.15–0.81)**	**0.17 (0.06–0.50)**
***ScEi***	0.44 (0.19–1.03)	**0.37 (0.14–0.98)**
***SiEc***	0.51 (0.21–1.19)	0.45 (0.18–1.14)
***SiEi***	**0.10 (0.02–0.52)**	0.19 (0.03–1.08)
***All conditions***	**0.35 (0.19–0.67)**	**0.30 (0.17–0.53)**
**Emotional**		
***ScEc***	0.58 (0.26–1.31)	0.76.(0.37–1.55)
***ScEi***	**0.18 (0.06–0.51)**	**0.37 (0.16–0.85)**
***SiEc***	0.52 (0.20–1.35)	**0.29 (0.11–0.78)**
***SiEi***	**0.21 (0.07–0.68)**	**0.11 (0.03–0.39)**
***All conditions***	**0.41 (0.22–0.75)**	**0.27 (0.14–0.52)**

The Table shows performance of patient groups as odds ratios (95% confidence intervals) referenced to healthy control group performance on the control tasks and auditory scene semantic and emotional congruity tasks; analyses of congruity test performance for each participant were based on scene stimuli containing sounds that were both individually identified correctly by that participant. Odds ratios with confidence intervals overlapping 1 indicate performance not significantly different from healthy controls; bold denotes significantly different from healthy controls (p < 0.05). ScEc, semantically congruous - emotionally congruous; ScEi, semantically congruous - emotionally incongruous; SiEc, semantically incongruous - emotionally congruous; SiEi, semantically incongruous - emotionally incongruous. bvFTD, patients with behavioural variant frontotemporal dementia; SD, patients with semantic dementia. Raw data are summarised for all tests and participant groups in Table S3 in Supplementary Material on-line.

#### 3.2.2 Auditory scene congruity decisions

**Fig. 2:**
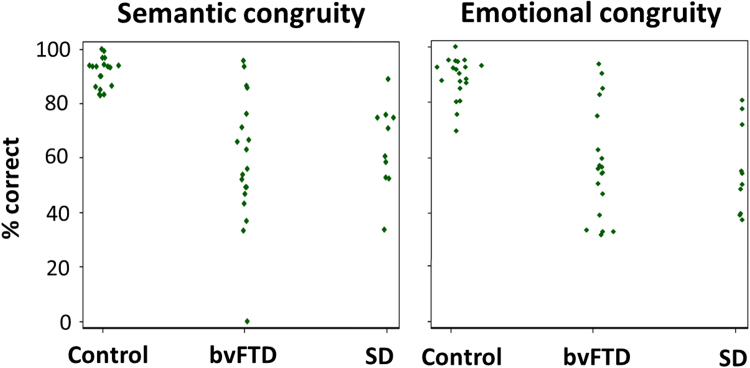
Raw group data for semantic and emotional congruity decisions on auditory scenes. Individual participant scores are plotted as proportion of trials correct for each auditory scene congruity task, for those scene stimuli comprising sounds that were both individually recognised correctly by that participant (note that there is therefore no ‘chance’ level of performance for these reduced data). bvFTD, patients with behavioural variant frontotemporal dementia; Control, healthy controls; SD, patients with semantic dementia.

Performance profiles of participant groups on the congruity decision tests are summarised in Table 2; individual raw scores are plotted in Fig. 2 and further details are provided in Table S3 in Supplementary Material on-line.

In the semantic scene congruity task (based on the scene stimulus subset with intact identification of constituent sounds, for each participant) there was an overall significant performance difference between participant groups (p < 0.0001). Both the bvFTD and SD groups performed significantly worse than healthy controls (p = < 0.001); there was no significant performance difference between patient groups nor evidence of an overall significant interaction between group and condition (p = 0.62).

In the emotional scene congruity task (based on the scene stimulus subset with intact identification of constituent sounds, for each participant), there was again an overall significant performance difference between participant groups (p = 0.0001), both the bvFTD and SD groups performing significantly worse than healthy controls in the congruous and incongruous conditions (all p < 0.005) with no significant performance difference between patient groups. There was no evidence of an overall significant interaction between group and condition (p = 0.14). However, the SD group trended toward a greater performance discrepancy between conditions than was shown by the healthy control group (p = 0.053). This effect was driven by relatively more accurate performance for scenes containing emotionally congruous sounds.

#### 3.2.3 Evaluation of auditory scene pleasantness

**Fig. 3:**
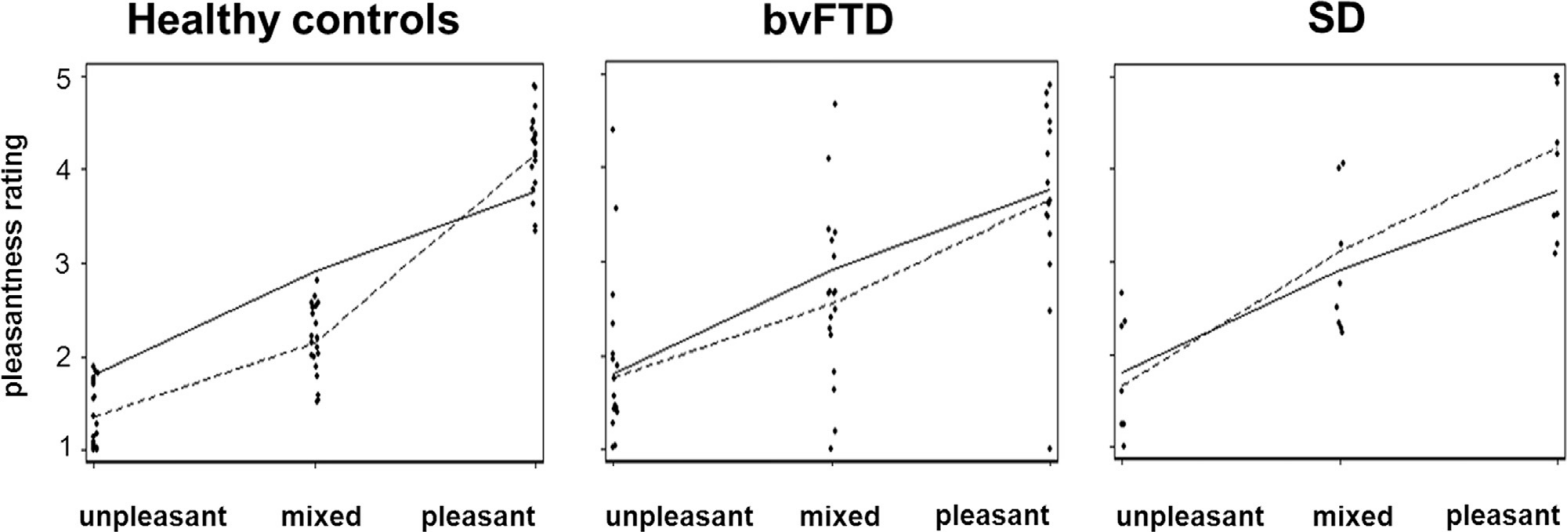
Individual data for rating pleasantness of auditory scene stimuli. For all individuals in each participant group, mean pleasantness ratings of auditory scene stimuli presented in the emotional congruity test (1, very unpleasant; 5, very pleasant) have been plotted against scene stimulus categories based on pilot healthy control group ratings of constituent sounds (**unpleasant**, pleasantness of both constituent sounds rated < 3; **mixed**, pleasantness of one sound > 3, other sound < 3; **pleasant**, pleasantness of both sounds > 3). On each plot, the solid line shows the calculated mean pleasantness rating of the two constituent sounds in each auditory scene, based on pilot healthy control group data; the dotted line shows the overall mean pleasantness of auditory scene stimuli in each category, as actually rated by participants in the main experiment. bvFTD, patients with behavioural variant frontotemporal dementia; SD, patients with semantic dementia.

Individual ratings of auditory scene pleasantness in the emotional congruity test are plotted in Fig. 3; further details of group profiles for rating the pleasantness of auditory scenes are presented in Table S5 in Supplementary Material on-line.

The SD group rated auditory scenes overall as significantly more pleasant than did the healthy control group (β = 0.73 [95% CI 0.25–1.29, p < 0.05]) while ratings of overall scene pleasantness by the bvFTD group did not differ significantly from healthy controls’ (β = 0.41 [95% CI −0.14 to 1.01, p > 0.05]); the two patient groups rated sound scenes similarly for overall pleasantness (β = 0.32 [95% CI −0.33 to 0.94, p > 0.05]).

The healthy control group exhibited an additive emotional effect of combining sounds into scenes (a significant positive interaction of sound pleasantness ratings) relative to individual sound pleasantness rated separately. Emotionally congruous auditory scenes were significantly more likely to be rated as pleasant than predicted from the individual sound ratings alone (β = 0.13 for interaction of sounds [95% CI 0.09–0.17, p < 0.05]; i.e., a 1 point increase in individual sound pleasantness rating was associated with an additional 0.13 point increase in scene pleasantness). This interaction effect was significantly stronger in healthy controls than in either patient group (for bvFTD vs controls, β = −0.09 [95% CI-0.15 to −0.003, p < 0.05]; for SD vs controls, β = −0.14 [95% CI −0.22 to −0.06, p < 0.05]). Indeed, neither patient group showed evidence of the effect (interaction of sounds in bvFTD, β = 0.05 [95% CI = −0.02 to 0.11, p > 0.05]; SD, β = −0.003 [95% CI = −0.07 to 0.07, p > 0.05]).

The healthy control group rated semantically congruous auditory scenes (within the emotional congruity test) as significantly more pleasant than semantically incongruous scenes (β = 0.15 [95% CI 0.05–0.26, p < 0.05]). This effect was replicated in the bvFTD group (β = 0.21 [95% CI 0.05–0.34, p < 0.05], but not in the SD group (β = 0.19 [95% CI −0.005 to 0.46, p > 0.05]). The effect was significantly stronger in healthy controls than in either patient group (for bvFTD, β = 0.05 [95% CI −0.14 to 0.22, p > 0.05]; for SD, β = 0.04 [95% CI −0.19 to 0.31, p > 0.05]) but did not differ significantly between patient groups (β = −0.01 [95% CI −0.26 to 0.28, p > 0.05]).

#### 3.2.4 Correlations between experimental and background measures

Accuracy of semantic and emotional auditory scene congruity decisions were significantly positively correlated in the bvFTD group (rho 0.80, p < 0.0001), but not the SD group (rho 0.54, p = 0.11). Accuracy of semantic scene congruity judgment and constituent sound identification (on the auditory semantic control task) were significantly positively correlated in the bvFTD group (rho 0.62, p = 0.005) but not the SD group (rho 0.55, p = 0.10). Semantic scene congruity judgment was significantly positively correlated with general executive capacity (WASI Matrices score) in the SD group (rho 0.91, p = 0.0002), though not the bvFTD group (rho 0.40, p = 0.09); with general semantic competence (BPVS score) in the bvFTD group (rho 0.49, p = 0.04) but not the SD group (rho 0.24, p = 0.51); and with a global measure of cognitive function (MMSE score) in both patient groups (bvFTD rho 0.50, p = 0.03; SD rho 0.79, p = 0.006). Emotional scene congruity judgment was significantly positively correlated with WASI Matrices score in the bvFTD group (rho 0.65, p = 0.003) but not the SD group (rho 0.47, p = 0.17); with BPVS score in both patient groups (bvFTD rho 0.45 p = 0.06; SD rho 0.79 p = 0.007); and with MMSE score in both patient groups (bvFTD rho 0.65, p = 0.004; SD rho 0.63, p = 0.049).

### 3.3 Neuroanatomical data

The patient groups showed the anticipated group-level, disease-related grey matter atrophy profiles: these encompassed bi-hemispheric prefrontal, anterior cingulate, insular and anterior temporal cortices and subcortical structures in the bvFTD group and leftward-asymmetric, predominantly antero-mesial temporal areas in the SD group (see Fig. S2 in Supplementary Material on-line).

**Fig. 4:**
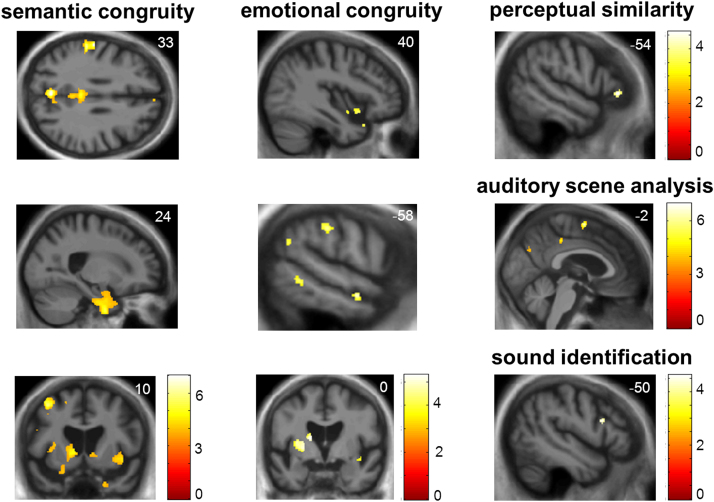
Neuroanatomical associations of auditory task performance in the patient cohort. The Figure shows statistical parametric maps (SPMs) of regional grey matter volume associated with performance on experimental tasks for the combined patient cohort, identified using voxel-based morphometry. Grey matter associations of semantic congruity processing in auditory scenes (left column), emotional congruity processing in auditory scenes (middle column) and auditory control tasks (right column) are presented (see text for details of contrasts). SPMs are overlaid on representative sections of the normalised study-specific T1-weighted mean brain MR image; the MNI coordinate (mm) of the plane of each section is indicated (the left cerebral hemisphere is shown on the left in the coronal sections and at the top in the axial section). Colour bars code T-score values for each SPM; SPMs are thresholded here at p < 0.001 uncorrected over the whole brain for display purposes, however regional local maxima were significant at p < 0.05_FWE_ corrected for multiple voxel-wise comparisons within pre-specified anatomical regions of interest (see Table 3).

Significant grey matter associations of behavioural measures for the combined patient cohort are summarised in Table 3 and statistical parametric maps of the behavioural correlates are presented in Fig. 4.

**Table 3:** Summary of neuroanatomical associations of auditory task performance in the patient cohort.

**Regional association**	**Area**	**Side**	**Cluster** (voxels)	**Peak** (mm)			**Z score**	**P value**
				x	y	z		
**SEMANTIC CONGRUITY**								
Parieto-temporal	*Precuneus*	*L*	*609*	*−3*	*−70*	*33*	*4.86*	*0.032*
	*SMG*	*L*	*757*	*−58*	*−20*	*33*	*4.83*	*0.036*
	PCC	L	59	−10	−58	22	4.51	0.005
		L	497	−6	−34	34	4.33	0.009
		R	276	2	−34	34	3.91	0.038
	Retrosplenial	L	27	−12	−42	4	4.15	0.017
	Post STG/STS	L	327	−57	−48	22	4.48	0.005
Ant temporal	Ant STS	L	100	−62	−6	−15	4.11	0.018
	Temporal pole	R	908	24	−2	−45	4.14	0.030
Insula	Ant insula	L	428	−34	2	−2	3.84	0.025
		R	546	38	18	−14	3.90	0.014
	Post Insula	R	65	39	−15	8	3.79	0.021
Pre-frontal	*Premotor*	*L*	*351*	*−39*	*14*	*54*	*4.79*	*0.042*
	mPFC/ACC	R	42	3	48	3	4.20	0.014
	IFG	L	160	−50	15	21	4.43	0.003
Striatum	Caudate head	L	409	−12	10	−2	3.82	0.045
**EMOTIONAL CONGRUITY**								
Ant temporal	Ant STS	L	52	−58	−9	−16	3.82	0.039
Insula	Ant insula	R	64	40	14	−14	3.49	0.046
Striatum	Putamen	L	709	−24	−2	3	4.07	0.017
	Caudate head	L		−15	0	14	4.07	0.018
**PERCEPTUAL SIMILARITY CONTROL**								
Pre-frontal	IFG	L	24	−54	34	−2	3.73	0.029
**AUDITORY SCENE CONTROL**								
Parieto-temporal	PCC	L	105	−10	−58	22	4.44	0.004
		R	99	2	−33	44	4.03	0.024
	Post STS	L	21	−66	−44	4	3.86	0.039
Pre-frontal	*SMA*	*L*	*182*	*−3*	*−3*	*64*	*4.85*	*0.034*
**SEMANTIC CONTROL (SOUND IDENTIFICATION)**								
Pre-frontal	IFG	L	29	−50	15	21	3.61	0.047

The Table shows grey matter associations of performance on experimental tasks for the combined patient cohort, identified using voxel-based morphometry. All local maxima exceeding significance threshold p < 0.05 after family-wise error correction for multiple voxel-wise comparisons, either over the whole brain (*italics*) or within pre-specified anatomical regions of interest (Supplementary Fig. S1) in clusters > 20 voxels in size are presented. Peak (local maxima) coordinates are in MNI standard space. Only positive grey matter associations are shown; no negative (inverse) associations were identified at the prescribed significance threshold. **ACC**, Anterior cingulate cortex; **Ant**, anterior; **IFG**, inferior frontal gyrus; **L**, left; **mPFC**, medial prefrontal cortex; **PCC**, posterior cingulate cortex; **Post**, posterior; **R**, right; **SMA,** Supplementary motor area; **SMG**, supramarginal gyrus; **STG**/**STS**, superior temporal gyrus/sulcus. See Section 2.2 for further details of experimental contrasts.

Impaired accuracy of judging the semantic congruity of auditory scenes was associated with grey matter loss in distributed, bi-hemispheric cerebral regions including precuneus, left supramarginal and premotor cortices (all p < 0.05_FWE_ corrected for multiple comparisons over the whole brain), posterior cingulate, posterior and anterior superior temporal, insular, medial prefrontal and inferior frontal cortices and caudate nucleus (all p < 0.05_FWE_ corrected for multiple comparisons within pre-specified anatomical regions). Impaired accuracy of judging the emotional congruity of auditory scenes was associated with grey matter loss in bi-hemispheric, anterior cortico-striatal areas including anterior superior temporal sulcus, insula, putamen and caudate nucleus (all p < 0.05_FWE_ corrected for multiple comparisons within pre-specified anatomical regions).

Significant grey matter associations were additionally identified for each of the experimental auditory control tasks. Accuracy of judging auditory perceptual similarity was associated with grey matter loss in left inferior frontal cortex. Impaired auditory scene analysis (impaired identification of spoken names from background babble) was associated with grey matter loss in prefronto-temporo-parietal regions including supplementary motor, anterior and posterior cingulate and posterior superior temporal cortices. Impaired sound identification was associated with grey matter loss in left inferior frontal cortex.

## 4 Discussion

Here we have shown that patients with bvFTD and SD have impaired processing of semantic and emotional congruence in auditory scenes relative to healthy older individuals. Both patient groups exhibited a similar profile of impaired congruence decisions about sound scenes. These deficits were evident after controlling for general executive, auditory semantic and auditory perceptual competence and not attributable to impaired identification or disordered affective valuation of individual constituent sounds. Taken together, our findings support the hypothesis that processing of auditory semantic and emotional relatedness is comparably impaired in both bvFTD and SD. Although there was no strong evidence overall for a specific condition effect, the SD group showed a tendency to more accurate determination of emotional congruity than incongruity in auditory scenes, suggesting a partial awareness of affective relatedness that was lost in the bvFTD group; in addition, performance in decoding the semantic and emotional congruity of auditory scenes was correlated in the bvFTD group but not the SD group, suggesting that the underlying processes are at least potentially dissociable. Previous work in SD and bvFTD has largely addressed the impaired semantic and affective coding of individual sensory objects, for which these syndromes show distinctive profiles of impairment. In contrast, the processing of semantic and affective relatedness might plausibly engage higher-order, associative and regulatory mechanisms, instantiated in extensive brain circuitry and jointly vulnerable in both syndromes. We therefore argue that the convergent deficits shown by our bvFTD and SD groups on these high-order semantic and affective tasks are consistent with previous studies of sensory object processing in these syndromes. The present findings corroborate a growing body of evidence for impaired processing of conflict and congruence in the auditory and other domains in bvFTD and SD, including striking impairments of socio-emotional signal decoding (Ahmed et al., 2014, Baez et al., 2014, Downey et al., 2015, Fletcher et al., 2016, Hughes et al., 2013, Ibanez and Manes, 2012, Irish et al., 2014, Krueger et al., 2009, Piwnica-Worms et al., 2010).

The present paradigm demonstrates a generic mechanism relevant to decoding of sensory signals in natural environments that might underpin a range of difficulties that patients both with bvFTD and SD experience in the more complex scenarios of daily life (for example, those surrounding ambiguous emotional communication, violation of social norms or conflicted moral choices (Carr et al., 2015; Downey et al., 2015; Eslinger et al., 2007; Kipps et al., 2009; Zahn et al., 2007)). Whereas defective detection of unexpected salient events would tend to promote the rigid and maladaptive behaviours that typify bvFTD and SD (Fumagalli and Priori, 2012, Snowden et al., 2003, Warren et al., 2013), inability to determine signal congruence could preclude the extraction of environmental regularities required for probabilistic learning and appropriate reward seeking (Dalton et al., 2012, Perry et al., 2014). Consistent with previous work (Krueger et al., 2009, Seer et al., 2015), the present study does not support a clear dissociation of congruence judgment from other aspects of executive function, but rather suggests this may be an ecologically relevant marker of failing executive processes. Nonverbal executive deficits have been shown to develop during the evolution of SD as well as bvFTD (Bozeat et al., 2000, Corbett et al., 2015, Gontkovsky, 2016, Smits et al., 2015). In this regard, it is of interest that the bvFTD group (but not the SD group) also showed a deficit on the auditory perceptual control task, in keeping with a more fundamental impairment of change detection or monitoring in this syndrome.

In addition to impaired cognitive decoding, as anticipated both the bvFTD and SD groups here showed altered affective valuation of auditory scenes. The SD group (though not the bvFTD group) tended to rate auditory scenes overall as more pleasant than did healthy controls. While this appears somewhat at odds with the high reported frequency of daily life sound aversion in this syndrome (Fletcher et al., 2015a), it is consistent with other evidence suggesting substantial modulation of affective responses by particular sounds in frontotemporal dementia syndromes (Fletcher et al., 2015b). More informative in the current context was the emotional effect of embedding sounds into scenes. Healthy controls rated emotionally congruous auditory scenes as more pleasant (and incongruous auditory scenes as less pleasant) than predicted from their own constituent individual sound ratings (Fig. 3, Table S5), whereas neither patient group showed evidence of this effect. In addition, healthy individuals rated semantically incongruous auditory scenes as less pleasant than congruous scenes: this effect was also evident (albeit attenuated) in the bvFTD group but not the SD group. In healthy individuals, affective integrative or ‘binding’ effects of combining emotional stimuli have been demonstrated previously in other modalities (Muller et al., 2011) and incongruity generally has increased aversive potential compared with congruity in various contexts (Piwnica-Worms et al., 2010, Schouppe et al., 2015). Information concerning the impact of neurodegenerative diseases on these processes remains very limited. The present findings suggest that both bvFTD and SD have impaired sensitivity to contextual modulation of affective signals, consistent with the more pervasive impairments of emotion processing documented in these syndromes (Kumfor and Piguet, 2012), whereas some sensitivity to the affective overtones of signal mismatch is retained in bvFTD but entirely lost in SD, consistent with the relative degree of semantic impairment in each syndrome.

The overlapping but partly separable neuroanatomical correlates of semantic and emotional congruity processing identified here suggest a framework for understanding the brain mechanisms that process different dimensions of auditory signal relatedness. These neuroanatomical substrates are in line with our experimental hypotheses and with previous neuroanatomical work in auditory and other modalities. Processing of both semantic and emotional auditory congruence had substrates in anterior temporal and insula cortices that are likely to constitute ‘hubs’ for processing signal patterns and salient deviations based on prior expectations or stored templates (Christensen et al., 2011, Clark et al., 2015b, Gauvin et al., 2016, Groussard et al., 2010, Merkel et al., 2015, Michelon et al., 2003, Nazimek et al., 2013, Remy et al., 2014, Watanabe et al., 2014). These regions are engaged during matching of incoming signals against previously learned semantic and affective schemas (Groussard et al., 2010, Zahn et al., 2009). The processing of auditory semantic congruence had additional correlates in distributed medial and lateral prefronto-parietal areas previously implicated in the processing of rule violations and reconciliation with previously established regularities, under a range of paradigms (Chan et al., 2012, Clark et al., 2015b, Gauvin et al., 2016, Groussard et al., 2010, Henderson et al., 2016, Jakuszeit et al., 2013, Michelon et al., 2003, Paavilainen, 2013, Pinhas et al., 2015, Remy et al., 2014, Ridderinkhof et al., 2004, Rosenbloom et al., 2012, Strelnikov et al., 2006, Watanabe et al., 2014).

The processing of auditory emotional congruence had an additional correlate in striatal structures broadly implicated in the evaluation of emotional congruence and reward (Dzafic et al., 2016, Klasen et al., 2011, Schultz, 2013). Although emotion and reward processing have classically been associated with ventral striatum rather than the dorsal striatal structures identified here, it is increasingly recognised that these striatal subregions participate in intimately integrated functional networks; moreover, dorsal striatum is particularly engaged during contingency monitoring and programming behavioural decisions on emotionally salient or incongruous stimuli (Haber, 2016).

A further potentially relevant issue is the lateralisation of cerebral regional atrophy profiles, which showed considerable variation across our patient cohort (Table 1). Based on other work in patients with right-- versus left-predominant temporal lobe atrophy (Binney et al., 2016, Kamminga et al., 2015), one might anticipate impaired processing of ‘rule-based’ semantic relatedness particularly in leftward asymmetric cases and impaired processing of affective relatedness in rightward asymmetric cases. As we adjusted for syndromic variation of atrophy profiles in our VBM analysis, it is unlikely that this factor confounded the neuroanatomical correlates observed. Moreover, previous work has also demonstrated that the temporal lobes participate jointly in a distributed semantic appraisal network and left- and right-lateralised presentations show extensive clinical overlap; it is therefore likely that substantially larger cohorts and functional neuroimaging techniques that can directly capture inter-hemispheric interactions will be required to resolve this issue.

The neural correlates of auditory semantic and emotional congruence decisions here overlapped with cortical associations of performance on the auditory control tasks, suggesting that these regions may be engaged as a functional network and that particular network components may play a more generic role in the analysis of stimulus relatedness. Performance on the auditory scene analysis control task had a substrate in temporo-parietal junctional and supplementary motor areas known to be fundamentally involved in parsing and monitoring of the auditory environment in healthy and clinical populations (Gauvin et al., 2016, Golden et al., 2015, Goll et al., 2012, Gutschalk and Dykstra, 2013, Zundorf et al., 2013). The temporo-parietal junction may serve as a domain-independent detector of salience associated with signal mismatch in diverse situations (Chan et al., 2012). Performance in both the perceptual similarity and sound identification control tasks here had a correlate in inferior frontal cortex: this region has been implicated previously in categorisation of sound stimuli particularly under conditions of high perceptual or cognitive load (Gauvin et al., 2016). The additional prefrontal, anterior temporal, insular and striatal correlates of auditory congruence processing identified here (see Table 3) might plausibly constitute domain-general substrates of signal relatedness decoding; again, however, this may only be substantiated by functional neuroimaging techniques that can assess communication between brain regions under different sensory modalities.

We regard this study as establishing proof of principle for the utility of the auditory congruence paradigm: the study has several limitations and suggests a number of directions for future work. Group sizes here were relatively small; studying larger cohorts would increase power to detect effects, particularly differences between syndromic groups (such as the bvFTD and SD groups here). The present findings have not established any strong specificity of auditory congruence deficits for particular neurodegenerative syndromes. There would be considerable interest in comparing these frontotemporal dementia syndromes with other syndromes and diseases, in order to assess the specificity of behavioural and neuroanatomical profiles of auditory signal relatedness processing for particular neurodegenerative pathologies. Alzheimer's disease, for example, might be expected to show a quite different profile of auditory conflict signalling based on available neuropsychological and neuroanatomical evidence (Fong et al., 2016). Equally pertinent will be longitudinal analyses to assess how the deficits identified here evolve over the course of illness, including presymptomatic stages in carriers of genetic mutations: core brain regions such as the insula have been shown to be involved prior to clinical symptom onset in genetic frontotemporal dementia (Rohrer et al., 2015) and behavioural correlation might yield a novel biomarker of imminent clinical conversion. In the world at large, signal integration and mismatch detection are rarely confined to a single sensory modality or time-point: multi- and cross-modal paradigms will likely amplify the findings here and it will also be of interest to assess the extent to which patients are able to learn new auditory ‘rules’ and adapt responses accordingly (Dalton et al., 2012, Michelon et al., 2003). Related to this, it will be relevant to assess the interaction of semantic and affective signal decoding, anticipated to drive much decision-making in real-world social exchanges (particularly the decoding of speech signals, as exemplified by sarcasm: Kipps et al., 2009). Structural neuroanatomical methods like those used here cannot capture dynamic processing and interactions between neural network components: future work should employ electrophysiological modalities with temporal resolution sufficient to track the dynamic signature of signal conflict and salience processing (Strelnikov et al., 2006) as well as connectivity-based anatomical techniques such as fMRI. Autonomic recordings would provide complementary information about the arousal potential of cognitive and affective decision-making on these auditory signals; this would likely help define disease phenotypes more fully (Fletcher et al., 2016, Fletcher et al., 2015b, Fong et al., 2016). Assessing the relevance of model systems of this kind will ultimately require correlation with clinical indices of socio-emotional functioning, which were not collected here.

Acknowledging the above caveats, this study suggests that auditory scene decoding may be a useful model paradigm for characterising the effects of dementias on signal processing in the more complex scenarios of daily life. From a clinical perspective, effective treatment of the dementias will likely depend on an accurate picture of the disability these diseases produce, in domains such as social and emotional cognition that are most sensitive to patients’ everyday functioning (St Jacques et al., 2015, Sturm et al., 2015); this in turn will require an informed deconstruction of complex, ill-defined symptoms to more tractable building blocks that can distil processes of clinical interest (Cicerone et al., 2006, Clark et al., 2015a). Our findings suggest that model auditory scenes can be constructed and manipulated relatively simply to achieve this. From a neuroanatomical perspective, we have shown that processing of signal relatedness in these simple auditory scenes engages the extensive brain circuitry of scene analysis, rule decoding and reward valuation. Targeting of large-scale intrinsic brain networks by neurodegenerative proteinopathies has proven to be a concept of considerable explanatory power (Zhou et al., 2010); the correlates of auditory scene decoding identified here do not respect conventional demarcations of the ‘salience’, ‘default-mode’ and other such networks. Rather, our data suggest that auditory semantic and emotional congruence analysis may depend on neural components distributed among intrinsically-connected networks. This interpretation is in line with an emerging paradigm emphasising network interactions in the processing of real-world, dynamic signal arrays that direct adaptive behaviours (Chiong et al., 2013). More speculatively, analysis of signal relatedness may engage a fundamental cognitive mechanism that is co-opted to the analysis of relatedness at different (sensory, perceptual, semantic, affective) levels of abstraction (Cohen, 2014). Template matching is one candidate universal algorithm that might support the necessary prediction testing, conflict detection and resolution in sensory systems (Friston, 2009); moreover, neural network architectures for template matching have been proposed and may be targeted by neurodegenerative pathologies (Clark and Warren, 2016, Warren et al., 2013). Key challenges for future work will be to establish whether sensory conflict and conguence signalling accesses a vulnerable neural architecture of this kind; and to determine whether this signal decoding paradigm can model the behavioural symptoms that blight patients’ daily lives.

## Disclosure

Nil conflicts of interest declared.

## Appendix A Supplementary material

Supplementary material

Supplementary material Ecsc = emotionally congruous semantically congruous.

Supplementary material Ecsi = emotionally congruous semantically incongruous.

Supplementary material Eisc = emotionally incongruous semantically congruous.

Supplementary material Eisi = emotionally incongruous semantically incongruous.
