# Supplementary material for: Auditory conflict and congruence in frontotemporal dementia
Source: Neuropsychologia. 2017 Sep;104:144–56. doi: 10.1016/j.neuropsychologia.2017.08.009 (PMC5637159; doi:10.1016/j.neuropsychologia.2017.08.009)
Supplement: Supplementary file 1 — Supplementary material [file mmc1.pdf]

SUPPLEMENTARY MATERIAL

Auditory conflict and congruence in frontotemporal dementia, by CN Clark et al

Details of stimulus synthesis. Sounds were selected from audio CDs (TMP computers sound effects, Digieffects, Warner Bros sound effects library (http://www.sound-ideas.coms) and a publically available on-line database: www.findsounds.com. Sounds were digitally resampled where necessary to a fixed rate of 44.1kHz and mean sound intensity level (root-mean-square (rms) value) was equated across individual sounds using Matlab7.0®. To form stimuli for the semantic and emotional congruency tests, pairs of sounds were then superimposed and edited to fix overall duration (8 seconds) and rms intensity value of the resulting auditory 'scenes' using Matlab7.0® and Goldwave®. Where necessary, brief or periodic sounds were concatenated and joined with intervening silent intervals to a total duration of 8 seconds. To form stimuli for the perceptual control test, individual (acoustically similar or dissimilar) sounds representing a given semantic category were concatenated to fixed duration (8 seconds) and rms level.

Examples of stimuli. Four audiofiles from the stimulus set have been included and are labelled as follows; ScEc_alarm_clock_snoring, Semantically congruous, emotionally congruous scene containing an alarm clock and snoring; ScEi_chiming_clock_snoring, Semantically congruous, emotionally incongruous scene containing a chiming grandfather clock and snoring; SiEc_alarm_clock_pig, Semantically incongruous, emotionally congruous scene containing an alarm clock and a pig grunting; SiEc_chiming_clock_lion, Semantically incongruous, emotionally congruous scene containing a chiming grandfather clock and roaring lion.

Pilot experiment. 10 healthy older individuals (six female; mean age 62.2 (s.d 3.9) years) participated in the pilot experiment. A series of 62 individual sounds was presented and participants were asked to identify each sound and to rate it using a Likert scale along dimensions of pleasantness (1 = very unpleasant, to 5 = very pleasant) and how alerting was the sound (1 = not alerting, to 5 = very alerting). The same sounds were also presented rearranged as superimposed pairs in 193 auditory 'scenes'; participants were asked to describe each scene and to rate on a Likert scale its overall pleasantness (1 = very unpleasant, to 5 = very pleasant) and how often the constituent sounds would be likely to be heard together (1 = very rarely, to 5 = very often).

An auditory scene was included in the final stimulus set if i) both constituent sounds were identified correctly by >80% of the pilot healthy control group and ii) the scene overall met an additional congruity criterion, based on pilot group ratings: for the semantic congruity test, likelihood of co-occurrence of the two sounds (semantically congruous, mean likelihood rating >3.5; semantically incongruous, mean likelihood rating <1.5) and for the emotional congruity test, pleasantness discrepancy of the two sounds (emotionally congruous, mean rated discrepancy <1; emotionally incongruous, mean rated discrepancy >2). In addition, scenes were selected such that each test was balanced wherever feasible for the 'nuisance' congruity parameter (for the semantic congruity test, the pleasantness discrepancy rating; for the emotional congruity test, the likelihood rating) and the individual sounds represented across conditions; and for the relative proportions of pleasant and unpleasant sound pairs comprising the congruous conditions.

Stimulus subsets after adjustment for individual sound identification. Restricting analyses to those auditory scene stimuli containing only correctly-identified individual sounds might potentially introduce a difficulty bias with respect to different participant groups. We therefore assessed whether the stimulus subsets analysed for each participant group differed systematically in scene likelihood or pleasantness between groups. For the subset of semantic scene stimuli

containing sounds that were both recognised individually, the average likelihood of the constituent sounds being found together (based on ratings by the healthy pilot group) did not differ significantly between participant groups (healthy controls, 3.08 (s.d.=1.40); bvFTD 3.05 (1.38); SD 3.08 (1.38); $p>0.05$). For this same stimulus subset, the average pleasantness discrepancy scores between the constituent sounds (based on pilot ratings) again did not differ between participant groups (healthy controls, 1.22 (1.05); bvFTD 1.20 (1.05); SD 1.23 (1.05); $p>0.05$). For the subset of emotional scene stimuli containing sounds that were both recognised individually, the average likelihood of the constituent sounds being found together (based on ratings by the healthy pilot group) did not differ significantly between participant groups (healthy controls, 3.41 (s.d. 1.41); bvFTD 3.40 (1.41); SD 3.36 (1.40); $p>0.05$). For this same stimulus subset, the average pleasantness discrepancy scores between the constituent sounds (based on pilot ratings) again did not differ between participant groups (healthy controls, 1.65 (1.37); bvFTD 1.66 (1.38); SD 1.69 (1.38); $p>0.05$).

Table S1. Auditory scene stimulus characteristics: semantic congruity test

Sound 1	Sound 2	Category	Likelihood	Pleasant 1	Pleasant 2	Discrepancy
Semantically congruous						
Gulls	Surf	ScEc	5	4.1	3.9	0.2
Train	Train crossing	ScEi	4.8	3.9	2.6	1.3
Babbling brook	Birds chirping	ScEc	4.7	4.4	4.3	0.1
Church organ	Church bell	ScEc	4.7	4.9	4.6	0.3
Baby laughing	Water splashing	ScEc	4.7	4.8	4.4	0.4
Sheep bleating	Rooster crowing	ScEc	4.7	4	3.6	0.4
Baby laughing	Carousel	ScEc	4.7	4.8	3.9	0.9
Sheep bleating	Pig grunting	ScEi	4.7	4	2.7	1.3
Alarm clock	Snoring	ScEc	4.5	2.4	1.8	0.6
Typewriter	Phone ringing	ScEc	4.3	3.6	3.2	0.4
Truck reversing	Pneumatic drill	ScEc	4.2	2.3	1.1	1.2
Water splashing	Baby crying	ScEi	4.1	4.4	1.4	3
Rain	Thunder	ScEi	3.8	3.3	2.3	1
Doorbell	Dog growling	ScEi	3.7	3.4	1.5	1.9
Clock chiming	Snoring	ScEi	3.7	4.3	1.8	2.5
		Mean	4.4	3.9	2.9	1.0
Semantically incongruous						
Woman crying	Pneumatic drill	SiEc	2	1.2	1.1	0.1
Wolf howling	Siren	SiEc	2	1.9	1.8	0.1
Wolf howling	Baby crying	SiEc	2	1.9	1.4	0.5
Cash register	Train crossing	SiEc	1.9	3	2.6	0.4
Surf	Typewriter	SiEc	1.8	3.9	3.6	0.3
Church bells	Lion roaring	SiEi	1.8	4.6	1.8	2.8
Alarm clock	Wolf howling	SiEc	1.7	2.4	1.9	0.5
Water splashing	Typewriter	SiEc	1.7	4.4	3.6	0.8
Applause	Scissors	SiEi	1.7	4.4	2.8	1.6
Baby laughing	Lion roaring	SiEi	1.7	4.8	1.8	3
Applause	Car alarm	SiEi	1.7	4.4	1.2	3.2
Applause	Pneumatic drill	SiEi	1.6	4.4	1.1	3.3
Pig grunting	Alarm clock	SiEc	1.5	2.7	2.4	0.3
Surf	Brushing teeth	SiEi	1.4	3.9	2.1	1.8
Clock chiming	Lion roaring	SiEi	1.3	4.3	1.8	2.5
		Mean	1.7	3.5	2.1	1.4

Sounds 1 and 2 are the constituent superimposed sounds composing each scene stimulus. Sound pairs are ordered here: primarily, in order of decreasing mean likelihood of co-occurrence (decreasing semantic congruity); secondarily, in order of increasing mean pleasantness discrepancy (decreasing emotional congruity), based on Likert ratings on a 5 point scale by the healthy pilot control group (scene stimuli were presented in randomised order during the test). Condition categories are designated as follows: ScEc, semantically congruous, emotionally congruous; ScEi, semantically congruous, emotionally incongruous; SiEc, semantically incongruous, emotionally congruous; SiEi, semantically incongruous, emotionally incongruous. Pilot group pleasantness ratings are shown for sound 1 and sound 2, respectively; the final column shows the modulus of the discrepancy rating for each pair.

Table S2. Auditory scene stimulus characteristics: emotional congruity test

Sound 1	Sound 2	Category	Likelihood	Pleasant 1	Pleasant 2	Discrepancy
Emotionally congruous						
Babbling brook	Birds chirping	ScEc	4.7	4.4	4.3	0.1
Dog growling	Cats fighting	ScEc	3.2	1.5	1.4	0.1
Woman screaming	Dentist's drill	SiEc	2.4	1.3	1.2	0.1
Woman crying	Pneumatic drill	SiEc	2	1.2	1.1	0.1
Gulls	Surf	ScEc	5	4.1	3.9	0.2
Baby laughing	Church bells	ScEc	3.9	4.8	4.6	0.2
Baby gurgling	Applause	ScEc	3.8	4.6	4.4	0.2
Siren	Glass breaking	ScEc	3.6	1.8	1.6	0.2
Woman screaming	Pneumatic drill	SiEc	1.9	1.3	1.1	0.2
Church organ	Church bell	ScEc	4.7	4.9	4.6	0.3
Baby gurgling	Clock chiming	ScEc	4.2	4.6	4.3	0.3
Duck quacking	Doorbell	SiEc	2.5	3.7	3.4	0.3
Dog growling	Dentist's drill	SiEc	2.3	1.5	1.2	0.3
Baby laughing	Water splashing	ScEc	4.9	4.8	4.4	0.4
Clock chiming	Train	SiEc	2.7	4.3	3.9	0.4
Lion roaring	Woman screaming	SiEc	1.9	1.8	1.3	0.5
Siren	Car alarm	ScEc	3.6	1.8	1.2	0.6
Lion roaring	Dentist's drill	SiEc	2.2	1.8	1.2	0.6
Lion roaring	Car alarm	SiEc	2.1	1.8	1.2	0.6
Wolf howling	Pneumatic drill	SiEc	1.9	1.9	1.1	0.8
		Mean	*3.1*	*2.9*	*2.6*	*0.3*
Emotionally incongruous						
Baby gurgling	Alarm clock	ScEi	3.6	4.6	2.4	2.2
Carousel	Baby crying	ScEi	4	3.9	1.4	2.5
Clock chiming	Snoring	ScEi	3.7	4.3	1.8	2.5
Birds chirping	Snoring	ScEi	3.5	4.3	1.8	2.5
Birds chirping	Siren	ScEi	3.4	4.3	1.8	2.5
Birds chirping	Lion roaring	ScEi	3.2	4.3	1.8	2.5
Baby laughing	Dog yelping	ScEi	3.7	4.8	2.1	2.7
Clock chiming	Baby crying	ScEi	3.5	4.3	1.4	2.9
Water splashing	Baby crying	ScEi	4.1	4.4	1.4	3
Applause	Cats fighting	SiEi	2	4.4	1.4	3
Baby laughing	Lion roaring	SiEi	1.7	4.8	1.8	3
Church bells	Dog growling	SiEi	2.1	4.6	1.5	3.1
Church bells	Baby crying	ScEi	3.4	4.6	1.4	3.2
Applause	Adult crying	SiEi	2	4.4	1.2	3.2
Applause	Car alarm	SiEi	1.7	4.4	1.2	3.2
Baby laughing	Dog growling	SiEi	2.2	4.8	1.5	3.3
Applause	Pneumatic drill	SiEi	1.6	4.4	1.1	3.3
Baby laughing	Dentist's drill	SiEi	2	4.8	1.2	3.6
Church bells	Vomiting	SiEi	1.8	4.6	1	3.6
Baby laughing	Vomiting	SiEi	1.8	4.8	1	3.8
		Mean	*2.8*	*4.5*	*1.5*	*3.0*

Sounds 1 and 2 are the constituent superimposed sounds composing each scene stimulus. Sound pairs are ordered here: primarily, in order of increasing mean pleasantness discrepancy (decreasing emotional congruity); secondarily, in order of decreasing mean likelihood of co-occurrence (decreasing semantic congruity), based on Likert ratings on a 5 point scale by the healthy pilot control group (scene stimuli were presented in randomised order during the test). Condition categories are designated as follows: ScEc, semantically congruous, emotionally congruous; ScEi, semantically congruous, emotionally incongruous; SiEc, semantically incongruous, emotionally congruous; SiEi, semantically incongruous, emotionally incongruous. Pilot group pleasantness ratings are shown for sound 1 and sound 2, respectively; the final column shows the modulus of the discrepancy rating for each pair.

Table S3. Performance of participant groups on auditory tasks: raw data

**Test**	**Healthy controls**	**bvFTD**	**SD**
**CONTROL TASKS**			
**Perceptual similarity** (/30)	0.91 (0.29)	**0.69 (0.46)**	0.83 (0.38)
**Auditory scene analysis** (/20)	0.98 (0.04)	**0.86 (0.12)**	**0.82 (0.20)**
**Sound identification** (/43)	0.96 (0.06)	**0.85 (0.36)**	**0.80 (0.40)**
**AUDITORY SCENE CONGRUITY Semantic**			
ScEc	0.93 (0.25)	**0.69 (0.46)**	**0.60 (0.49)***
ScEi	0.85 (0.36)*	0.54 (0.50)	**0.56 (0.50)***
SiEc	0.89 (0.31)*	0.66 (0.48)	0.70 (0.46)*
SiEi	0.98 (0.15)	**0.68 (0.47)**	0.82 (0.39)
All conditions	0.92 (0.28)	**0.65 (0.48)**	**0.67 (0.47)**
Total stimuli analysed (presented)	596 (600)	447 (570)	247 (300)
**Emotional**			
ScEc	0.87 (0.34)*	0.65 (0.48)	0.74 (0.44)*
ScEi	0.87 (0.33)*	**0.55 (0.50)**	**0.44 (0.50)***
SiEc	0.86 (0.35)*	0.60 (0.49)	**0.53 (0.50)**
SiEi	0.94 (0.23)	**0.64 (0.48)**	**0.55 (0.50)**
All conditions	0.89 (0.32)	**0.61 (0.49)**	**0.57 (0.50)**
Total stimuli analysed (presented)	792 (800)	594 (760)	331 (400)

Mean group raw scores are shown as proportion correct (standard deviation) for each auditory test. Maximum scores are indicated (in parentheses) for the auditory control tests. Congruity tests were scored for each participant on those scene stimuli containing sounds that were both identified correctly by that participant when presented in isolation in the auditory semantic control test; the total numbers of stimuli included in this subanalysis for each congruity task and each participant group are shown (total number of stimuli presented to that group of participants in parentheses; individual participants all heard the full set of stimuli). For the perceptual similarity task (two-alternative forced choice), a chance score corresponds to 0.5; for the sound identification task (three alternative forced choice), a chance score corresponds to 0.33. *denotes significantly different from reference condition (SiEi); bold denotes significantly different from healthy control group. ScEc, semantically congruous - emotionally congruous; ScEi, semantically congruous - emotionally incongruous; SiEc, semantically incongruous - emotionally congruous; SiEi, semantically incongruous - emotionally incongruous. bvFTD, patients with behavioural variant frontotemporal dementia; SD, patients with semantic dementia. See text for details of tests

Table S4. Pleasantness ratings of individual sounds for each participant group

Sound	Healthy controls	bvFTD	SD
Babbling brook	4.75	4.16	3.50
Birds chirping	4.65	3.95	3.60
**Water splashing**	**4.65**	**3.68**	**3.50**
Baby gurgling	4.60	3.74	3.80
Surf	4.60	3.63	3.70
Baby laughing	4.50	3.58	4.50
Church organ	4.30	4.16	4.60
Church bells	4.20	3.74	4.40
Sheep bleating	4.20	2.95	3.20
Carousel	4.15	3.63	4.30
Duck quacking	4.15	3.26	3.60
Rooster crowing	4.00	3.05	3.90
Applause	3.95	3.26	4.20
Clock chiming	3.95	3.47	4.00
Pig grunting	3.80	2.95	3.20
Gulls	3.75	3.32	3.50
Train	3.75	3.21	4.00
Doorbell	3.40	3.05	3.90
Rain	3.35	2.63	3.20
Typewriter	3.30	2.79	3.30
Thunder	3.25	2.58	2.70
Phone ringing	3.15	3.53	3.60
Scissors	3.05	2.89	3.40
Train crossing	3.05	3.05	3.10
Cash register	2.95	2.74	2.60
Brushing teeth	2.95	2.63	3.60
Lion roaring	2.75	2.68	3.00
Wolf howling	2.55	2.68	3.10
Dog yelping	2.35	2.74	3.20
Alarm clock	2.25	2.68	3.30
Snoring	2.20	1.74	2.50
Truck reversing	2.10	2.74	2.90
**Siren**	**2.10**	**3.00**	**3.30**
Baby crying	1.95	2.00	2.70
Pneumatic drill	1.80	2.11	2.80
Cats fighting	1.60	2.21	2.10
Glass breaking	1.55	2.16	1.90
**Car alarm**	**1.50**	**2.42**	**3.00**
Dog growling	1.50	1.84	2.70
Dentist's drill	1.50	2.21	2.50
**Adult crying**	**1.10**	**1.58**	**2.40**
Vomiting	1.10	1.47	1.90
**Woman screaming**	**1.05**	**1.68**	**2.00**

Mean pleasantness ratings for each participant group for the individual sounds used to create auditory scene stimuli are shown. Sounds have been ordered here from most to least pleasantly rated by the healthy control group. Those sounds for which patient groups both showed a significant discrepancy from healthy control ratings (based on detection of an overall group effect using Kruskal-Wallis and between-group comparison using Wilcoxon rank sum tests, $p<0.05$) are indicated in bold. bvFTD, behavioural variant frontotemporal dementia; SD, semantic dementia.

Table S5. Comparison of participant groups for rating pleasantness of auditory scene stimuli

Group	Scene pleasantness					Constituent sound pleasantness effect			
	**ScEc**	**ScEi**	**SiEc**	**SiEi**	**Overall**	*Within group*		*Control comparison*	
						Coeff	95% CI	Coeff	95% CI
Control	3.38 (1.45)	2.45 (0.84)	1.82 (1.02)	2.01 (0.80)	2.4 (1.2)	**0.13**	**0.09 to 0.17**	-	-
bvFTD	3.32 (1.50)	2.65 (1.28)	2.33 (1.39)	2.49 (1.24)	2.7 (1.4)	0.05	-0.02 to 0.11	**-0.09**	**-0.15 to -0.003**
SD	3.67 (1.47)	3.14 (1.37)	2.60 (1.49)	3.26 (1.41)	**3.2 (1.5)**	-0.002	-0.07 to 0.07	**-0.14**	**-0.22 to -0.06**

For each participant group, raw mean (standard deviation) scene pleasantness ratings of all auditory scene stimuli administered in the emotional congruity test are shown by sound scene condition (left) together with coefficients (Coeff) and 95% confidence intervals (CI) for the effect of interacting constituent sound pleasantness ratings, within each group and for patient groups relative to the reference healthy control group (right; see 3.2 for details). A confidence interval crossing 0 indicates no significant difference relative to the healthy control group; bold indicates significantly different from healthy controls ($p<0.05$). ScEc, semantically congruous - emotionally congruous; ScEi, semantically congruous - emotionally incongruous; SiEc, semantically incongruous - emotionally congruous; SiEi, semantically incongruous - emotionally incongruous; Control, healthy control group; bvFTD, patients with behavioural variant frontotemporal dementia; SD, patients with semantic dementia.

Figure S1

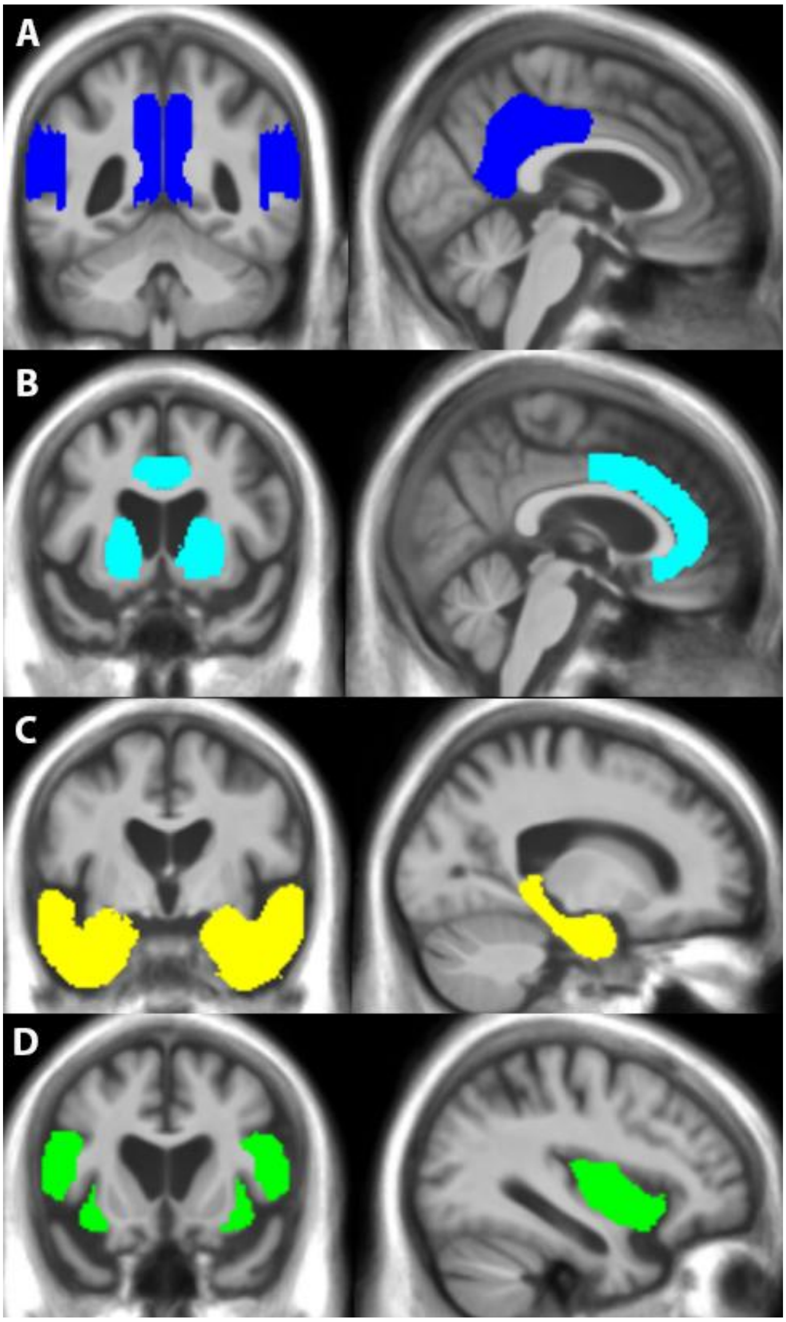

Figure S1. Representative coronal (left) and sagittal (right) sections of anatomical regions used for multiple voxel-wise comparisons correction in region-of-interest analyses based on prior anatomical hypotheses (see section 2.6). Bi-hemispheric regions of interest are rendered on sections of the average normalised brain template for the combined patient cohort; the left cerebral hemisphere is shown on the left in coronal sections. Regions comprised: **A,** lateral temporo-parietal and posterior medial cortices (auditory scene perception); **B,** striatum and anterior cingulate cortex (salience and reward evaluation); **C,** anterior and medial temporal lobe (semantic processing); **D,** insula and inferior frontal gyrus (auditory sequence and rule-based processing). In line with prior anatomical hypotheses, volume A was assessed in all experimental contrasts; while volumes B, C and D were assessed in contrasts based on auditory semantic and/or congruity processing (i.e., all contrasts apart from the auditory scene control contrast).

Figure S2

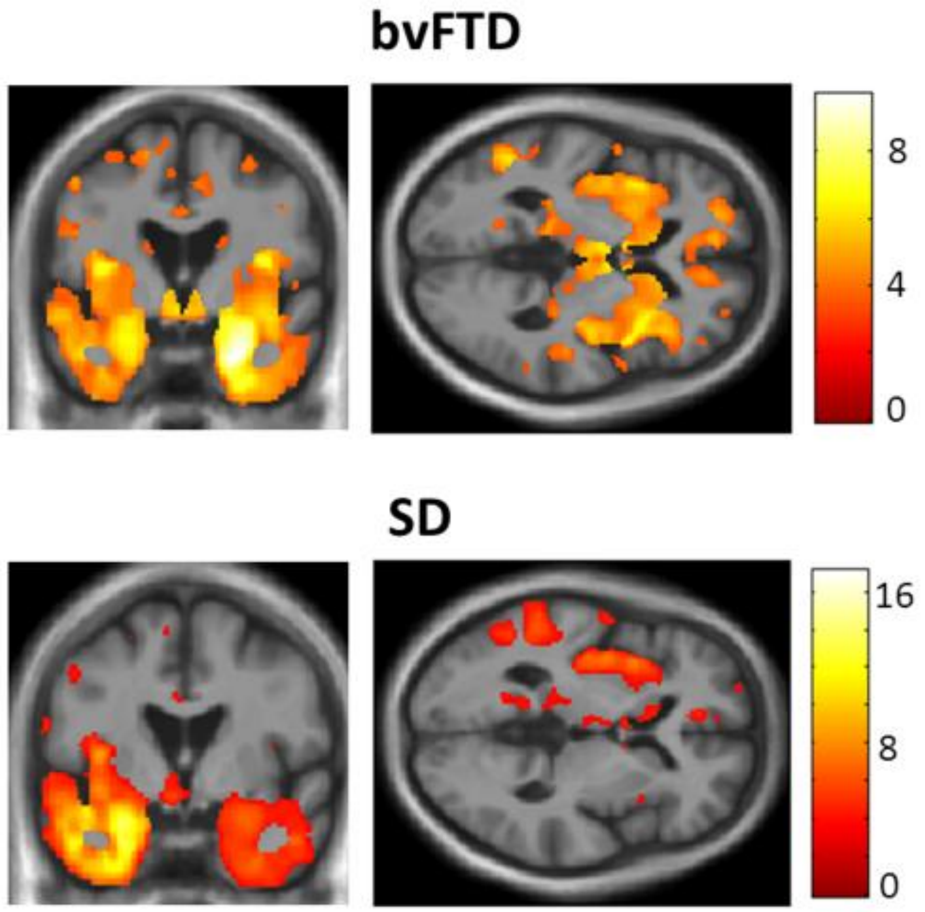

Figure S2. Profiles of disease-related grey matter atrophy in the patient groups. Statistical parametric maps (SPMs) show regions of significant grey matter reduction in the behavioural variant frontotemporal dementia (bvFTD) group and the semantic dementia (SD) group compared to the present healthy control group, identified using voxel-based morphometry. SPMs are overlaid on representative coronal (left) and axial (right) sections of a group mean T1-weighted MR image in MNI space, thresholded leniently for display purposes at $p<0.01$ uncorrected over the whole brain. The colour bars code T-score values for each SPM; the left cerebral hemisphere is shown on the left in the coronal sections.
